# Location‐Dependent Lanthanide Selectivity Engineered into Structurally Characterized Designed Coiled Coils

**DOI:** 10.1002/anie.202110500

**Published:** 2021-10-07

**Authors:** Louise N. Slope, Oliver J. Daubney, Hannah Campbell, Scott A. White, Anna F. A. Peacock

**Affiliations:** ^1^ School of Chemistry University of Birmingham Edgbaston B15 2TT UK; ^2^ School of Biosciences University of Birmingham Edgbaston B15 2TT UK

**Keywords:** bioinorganic chemistry, coiled coils, lanthanides, peptides, protein design

## Abstract

Herein we report unprecedented location‐dependent, size‐selective binding to designed lanthanide (Ln^3+^) sites within miniature protein coiled coil scaffolds. Not only do these engineered sites display unusual Ln^3+^ selectivity for moderately large Ln^3+^ ions (Nd to Tb), for the first time we demonstrate that selectivity can be location‐dependent and can be programmed into the sequence. A 1 nm linear translation of the binding site towards the N‐terminus can convert a selective site into a highly promiscuous one. An X‐ray crystal structure, the first of a lanthanide binding site within a coiled coil to be reported, coupled with CD studies, reveal the existence of an optimal radius that likely stems from the structural constraints of the coiled coil scaffold. To the best of our knowledge this is the first report of location‐dependent metal selectivity within a coiled coil scaffold, as well as the first report of location‐dependent Ln^3+^ selectivity within a protein.

## Graphical abstract

Location‐dependent size selectivity can be programmed into designed metallo coiled coils, allowing for the generation of near‐identical selective and promiscuous metal‐ion sites. The first crystal structure of a lanthanide bound within a coiled coil reveals that the existence of an optimal radius stems from the structural constraints of the peptide scaffold. Therefore, a selective site can in principle be designed de novo for any given Ln^3+^ ion.

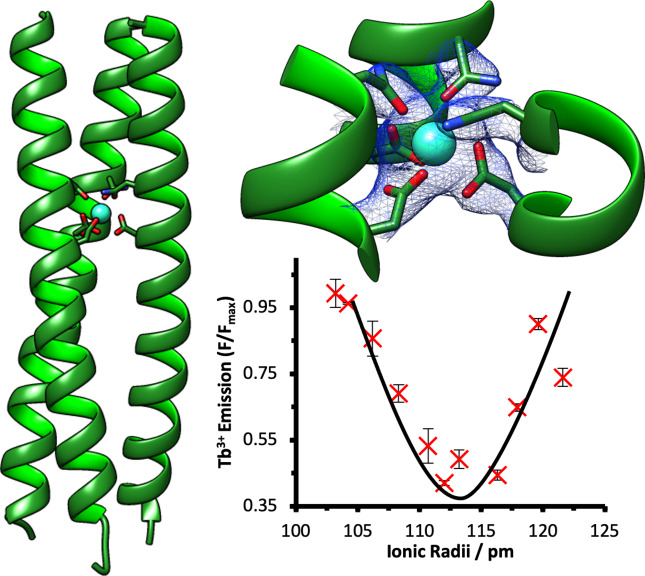

The artificial design of functional metalloproteins is extremely exciting given that metal ion sites perform a vast range of essential biological roles, including: acting as catalysts, participating in electron transfer, and stabilizing protein structure.^[1]^ Many designed metalloproteins are based on coiled coils, a class of miniature protein scaffolds which lack the complexity of native proteins and into which biomimetic metal ion sites are increasingly being engineered,[^1^, ^2^] often providing unique insight into metal ion biochemistry. An attractive alternative approach is to engineer rare or xenobiotic ion sites that offer novel function, chemistries and opportunities beyond the repertoire of biology.^[3]^ An appealing class of ions are the lanthanides, which have only relatively recently been identified as being biologically essential metals.^[4]^ Nature has taken advantage of the high Lewis acidity of the lanthanides at enzyme active sites. In contrast, chemists exploit the full range of appealing chemical properties of this class of metal ions. Many lanthanide ions emit at defined wavelengths including visible, infrared and near‐infrared, with narrow emission lines and long emission lifetimes. Other lanthanides are paramagnetic, and find applications in NMR and as magnetic resonance imaging (MRI) contrast agents.^[5]^

The attractive photophysical and magnetic properties of Ln^3+^ sites are such that they have been widely introduced into native proteins artificially, and, to a lesser extent, into compact protein motifs including a de novo designed TIM barrel^[6]^ and coiled coil structures.^[7]^ We reported the design of the first gadolinium coiled coil, which displayed superior MRI relaxivity at 7 T to that of small‐molecule complexes used in the clinic.^[7f]^ Our design (MB1‐2) features an asparagine (Asn, N), aspartate (Asp, D) hard oxygen donor binding site within the hydrophobic core of a parallel three‐stranded coiled coil (Asn‐Xxx_3_‐Asp)_3_, and was found to bind Tb^3+^, Ce^3+^, Nd^3+^, Eu^3+^, Dy^3+^, Er^3+^ and Yb^3+^, in addition to Gd^3+^.^[7f]^ This binding site can be linearly translated at 1 nm intervals along the coiled coil, creating an isomeric series of peptides (Table 1 and Figure 1), and though Ln^3+^ binding is retained across this series, the coordination chemistry is not.^[7g]^ For example, a single heptad binding site translation from the middle to the N‐terminus, transforms a coordinatively saturated Tb(Asn‐Xxx_3_‐Asp)_3_ site, into a highly hydrated Tb(Asp)_3_(H_2_O)_3_ site.^[7g]^ Design rules established with simpler coiled coil scaffolds would have important implications for 1) the design of functional Ln^3+^ coiled coils and metallocoiled coils more widely, and 2), they could provide important insight into native lanthanide biochemistry.

**Figure 1:**
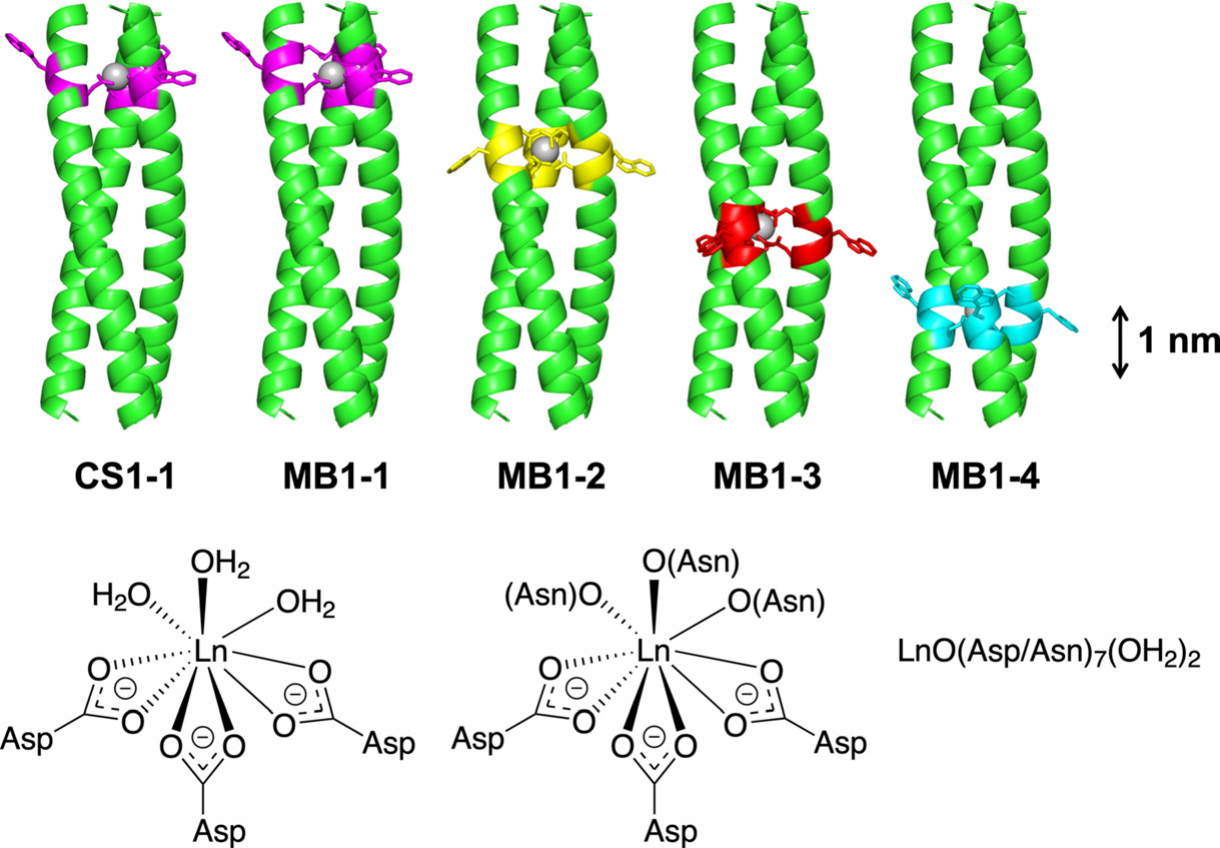
Models of the Ln^3+^ coiled coils used in this study. The main‐chain atoms are represented as helical ribbons with the N‐terminus at the top, binding sites are highlighted, coordinating residues and Trp side‐chains are shown in stick form, and the Ln^3+^ ion as a sphere. ChemDraw structures show the Ln^3+^ binding sites associated with CS1‐1/MB1‐1 and MB1‐2/MB1‐3. Ln^3+^ binding to MB1‐4 involves the coordination of two water molecules and a mixture of Asp/Asn donors.

**Table 1:** Peptide sequences used in this work.

Peptide	Sequence^[a]^ (N→C terminus)
*heptad*	Ac‐*g abcdefg abcdefg abcdefg abcdefg abcdefg a*
CS1‐1	Ac‐G IAAIE **W** K **D** AAIEQK IAAIEQK IAAIEQK IAAIEQK G‐NH_2_
MB1‐1	Ac‐G IAA **N** E **W** K **D** AAIEQK IAAIEQK IAAIEQK IAAIEQK G‐NH_2_
MB1‐2	Ac‐G IAAIEQK IAA **N** E **W** K **D** AAIEQK IAAIEQK IAAIEQK G‐NH_2_
MB1‐3	Ac‐G IAAIEQK IAAIEQK IAA **N** E **W** K **D** AAIEQK IAAIEQK G‐NH_2_
MB1‐4	Ac‐G IAAIEQK IAAIEQK IAAIEQK IAA **N** E **W** K **D** AAIEQK G‐NH_2_
HC02	Ac‐E WEAIEKK IAA **N** ESK **D** QAIEKK IQAIEKK IEAIEHG‐NH_2_

[a] Binding site residues, and where relevant the adjacent Trp sensitizer, are in bold and underlined.

The similar coordination behavior across the lanthanide series makes it extremely challenging to discriminate between Ln^3+^ ions,^[8]^ but there is a real desire to be able to do so given their different magnetic and photophysical properties.^[9]^ One opportunity for discrimination is provided by the reduction in ionic radius as you move from left to right across the lanthanide series. Lanthanide‐dependent dehydrogenases show a dependence on the larger, more abundant Ln^3+^ ions,^[10]^ as does the lanthanide‐ binding protein Lanmodulin featuring Ln^3+^ selective EF‐hand like sites^[4d]^ and the growth of lanthanide‐ utilising bacteria.^[11]^ In contrast, the competitive displacement of bound Tb^3+^ ions to native Ca^2+^ EF‐hand like sites displays a bias towards the small, charge‐dense Ln^3+^ ions.^[12]^ Lanthanide‐binding peptide tags show a small preference for intermediate ions (Eu^3+^ to Er^3+^),^[13]^ and a “lanthanide finger” protein is complementary for the Tb^3+^ to Er^3+^ size range.^[7h]^ Although Ln^3+^ coiled coils display selectivity for Ln^3+^ ions over Ca^2+^,[^7c^, ^7d^, ^7e^, ^7f^] there have been no studies relating to selectivity across the lanthanide series. Given the desire to distinguish between the different lanthanide ions, and the reports of coiled coils capable of metal ion discrimination based on size,^[14]^ the presence of lone pairs,^[15]^ and hard soft acid base theory,[^2a^, ^16^] we set out to establish whether size‐selective Ln^3+^ binding sites can be engineered into coiled coils, and to obtain crucial, and currently lacking, structural information regarding Ln^3+^ coiled coils.

In an effort to identify Ln^3+^‐selective sites we performed a systematic study on a series of coiled coils featuring Ln^3+^‐binding sites (Table 1 and Figure 1), and by investigating twelve different Ln^3+^ ions, have for the first time demonstrated location‐dependent Ln^3+^ selectivity.

As our designs feature a tryptophan (Trp) adjacent to the Ln^3+^‐binding site capable of sensitising Tb^3+^ luminescence, we performed Tb^3+^ displacement experiments with eleven Ln^3+^ ions, which has been widely adopted in the literature to establish Ln^3+^‐binding preferences to Ca^2+^‐binding proteins and Ln^3+^‐binding derivatives thereof.[^12^, ^17^] One molar equivalent of Tb^3+^ was added to each of the five coiled coils (30 μM monomer, 10 μM trimer), each containing a different binding site, and sensitised emission was detected upon excitation of the Trp at 280 nm, thereby limiting interference from any free Tb^3+^ in solution. A decrease in the characteristic Tb^3+ 5^D_4_ to ^7^D_5_ emission at 545 nm can be used as a measure of Tb^3+^ displacement by competing Ln^3+^ ions (Eu^3+^ emission, also sensitised by Trp,^[18]^ does not overlap in this range), and was quantified as the ratio of the emission in the presence and absence of competing metal, *F*/*F*_max_. Spectra were recorded directly following sample preparation (ca. 15 minutes), as well as following 24 and 72 hours equilibration in 10 mM HEPES buffer pH 7.0 (Figures 2, S2, S3 and S4), and are consistent with equilibration within the initial 15 minutes. Analogous displacement experiments were conducted at pH 5.5 (30 mM MES buffer) given potential issues associated with the formation of lanthanide hydroxide species above pH≈6.5 (Figure S4C).^[19]^ Similarly, for MB1‐2, displacement experiments were conducted at the higher concentration of 100 μM MB1‐2 monomer, to explore concentration dependence and issues around optimal metal induced folding (Figure S5). However, in all cases, these experiments yielded similar displacement profiles.

**Figure 2:**
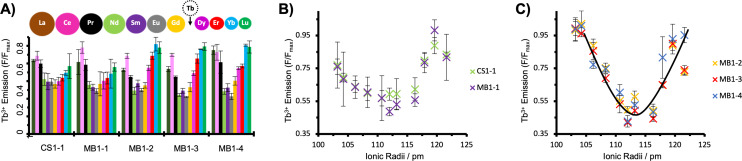
A) Luminescence Tb^3+^ displacement plots for 10 μM Tb^3+^ and 30 μM peptide monomer in the presence of 10 μM competing Ln^3+^ ion, in 10 mM HEPES buffer pH 7.0 following 72 hours equilibration. Spheres are shown as an indication of the change in Ln^3+^ size across the series. Dependence of Tb^3+^ displacement on the effective ionic radii of the Ln^3+^ ions, for B) terminal sites (CS1‐1, MB1‐1) and C) for the central and C‐terminal sites (MB1‐2, MB1‐3 and MB1‐4). Data are based on the integration of the 545 nm Tb^3+^ emission peak, for experiments performed in triplicate, and bars represent the standard deviation. The line shown indicates the apparent trend in panel C), but does not reflect a true fit.

The addition of one equivalence of Gd^3+^, which is often used interchangeably with Tb^3+^ due to its similar ionic radius,[^7f^, ^7g^] leads to roughly half of the Tb^3+^ being displaced from each of the five binding sites as expected (Figure 2 and Figure 3). Similar displacement was observed for experiments conducted with medium‐sized competing ions Eu^3+^, Sm^3+^ and Nd^3+^, despite the increase in size, and indicates that the designed site is unable to discriminate between Ln^3+^ ions within this size‐range (109.5–116.3 pm, for 9‐coordinate complexes of Tb^3+^‐Nd^3+^),^[20]^ regardless of binding‐site location and coordination chemistry.

**Figure 3:**
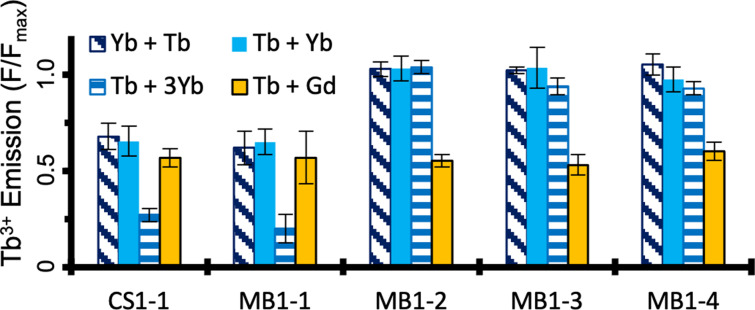
Luminescence Tb^3+^ displacement plots for 10 μM Tb^3+^ and 30 μM peptide monomer, on addition of one (block blue) or three (horizontal lines) equivalents of Yb^3+^, on changing the order of addition (diagonal lines) and on addition of one equivalent of competing Gd^3+^ (block orange), in 10 mM HEPES buffer pH 7.0 following 72 hours equilibration. Data are based on integration of the 545 nm Tb^3+^ emission peak, for experiments performed in triplicate, and bars represent the standard deviation.

When the competing ion is larger, as is the case with Ce^3+^, Pr^3+^ and La^3+^, there is a notable decrease in Tb^3+^ displacement (Figures 2 and S2–S5), in some cases consistent with the large majority of Tb^3+^ remaining bound. This is largely similar for all five binding sites, including those located towards either the N‐ or C‐terminus (CS1‐1, MB1‐1 and MB1‐4) which one might envision are more frayed and flexible. Intriguingly, this behavior does not entirely correlate with ionic size, with Ce often less effective at displacing Tb compared to its larger neighbour La. Ce, with its richer redox chemistry and accessible +4 oxidation state, was also reported to bind differently to the other lanthanides to a designed TIM barrel protein.^[6]^

When the competing ion is smaller in size than Tb^3+^, a stark contrast in behavior between the five different binding sites is observed (Figures 2, 3 and S2–S5). For those sites located towards the N‐terminus (as is the case for MB1‐1 and CS1‐1), no significant discrimination is evident when the competing Ln^3+^ ion is a smaller Dy^3+^, Er^3+^, Yb^3+^ or Lu^3+^ ion. In contrast, the remaining three MB1 peptides in which the (Asn‐Xxx_3_‐Asp)_3_ binding site is located more centrally or towards the C‐terminus (MB1‐2, MB1‐3 and MB1‐4), discriminate against these smaller ions in a size‐dependent fashion (Figures 2 and S2–S5). Notably, the equilibrium position in the presence of Lu^3+^ and Yb^3+^ ions is consistent with Tb^3+^ bound to these three binding sites under these experimental conditions, and when presented with intermediate‐sized Ln^3+^ ions, such as Dy^3+^ or Er^3+^, partial displacement is observed (Figures 2 and S2–S5). This discrimination based on size, is akin to the ability of ion channels to discriminate between otherwise similar metals, Na^+^ and K^+^.^[21]^

Given that Yb^3+^ is unable to displace Tb^3+^ from three of the five binding sites, we explored whether this could be driven by the addition of excess Yb^3+^. However, even three equivalents of Yb^3+^ does not lead to notable displacement of Tb^3+^ from the selective sites (MB1‐2, MB1‐3 and MB1‐4). In contrast, the non‐selective N‐terminal sites (CS1‐1 and MB1‐1) sites showed further Tb^3+^ displacement, consistent with their inability to discriminate effectively between these two ions (Figure 3). To rule out a kinetic effect, both the order of addition and the impact of equilibration time, were investigated. In both cases, the results were found to be the same within error (Figures 3 and S2–S5).

Taken together, these findings suggest that when located towards the N‐terminus, regardless of whether the binding site is generated by a single (Asp)_3_ layer, or a double (Asn‐Xxx_3_‐Asp)_3_ site, local structural changes such as fraying, generate a more flexible and malleable binding site,[^22^, ^23^] which can accommodate metals of a wider range of sizes. In contrast, the remaining binding sites are more rigid and less deformable, being more centrally located along the coiled coil. At first it might appear surprising that the MB1‐1 and MB1‐4 binding sites behave so differently, as both are situated towards the ends of the coiled coil. However, the binding site location and L‐stereochemistry of the amino acids leads to two non‐identical binding sites,^[7g]^ with the MB1‐4 site located more centrally within the coiled coil. The structural constraints of binding sites buried within a three‐stranded coiled coil are such that an optimal Ln^3+^ radius for binding falls between that of Nd^3+^ and Tb^3+^.

To the best of our knowledge this is the first report in which metal selectivity within a coiled coil scaffold is location‐dependent, as well as the first report of location‐dependent Ln^3+^ selectivity within a protein scaffold more generally. Furthermore, it should be possible to tailor‐design sites for a specific Ln^3+^ radius through careful selection of coiled coil oligomeric state, or through binding site redesign.

To elucidate the structural origin of Ln^3+^ selectivity, a crystal structure of an optimally sized Tb^3+^ bound to the designed (Asn‐Xxx_3_‐Asp)_3_ site in the size‐selective central binding location was obtained. Attempts to crystallise the MB coiled coils repeatedly proved unsuccessful, so an analogous coiled coil scaffold more amenable to crystallisation was adopted, based on CoilSer (CS) and the longer derivative GRAND‐CS, designed by DeGrado and Pecoraro, respectively.[^24^, ^25^] The external residues, which are likely critical for the formation of favourable crystal packing interactions, were retained, but the core was replaced with that of MB1‐2. Thus the Leu hydrophobic core of CS was replaced with Ile, and our Asn and Asp binding‐site residues were introduced into positions 12 and 16. The resulting coiled coil, HC02 (Table 1), displays similar Tb^3+^ binding to that of MB1‐2 (Figure S6).

Crystals of the Tb^3+^ complex were obtained in the *H*3 space group and the structure solved to 2.1 Å resolution, to yield a parallel three‐stranded coiled coil with a clearly identifiable Tb^3+^ (based on strong anomalous scattering) bound in its centre between adjacent Asp and Asn layers (Figures 4 and S7, and PDB 7P3H). To the best of our knowledge this is the first crystal structure of a Ln^3+‐^binding site engineered within the hydrophobic core of a coiled coil, and provides excellent confirmation of our designed (Asn‐Xxx_3_‐Asp)_3_ binding site.^[26]^ The refined structure is consistent with all Tb‐O distances for the binding‐site residues falling between 2.3 and 2.6 Å, in good agreement with previous peptide/protein complexes (Table S2).[^4^, ^6^, ^11^, ^13^, ^26^, ^27^] Though the electron density cannot assign whether it is the Asn nitrogen or oxygen that is bound, the Tb‐X distances are in better agreement with Tb‐O than the longer Tb‐N distances reported in the literature.^[28]^ With the exception of the terminal residues, involved in the formation of favorable crystal packing interactions facilitated by external Tb^3+^/Zn^2+^, all residues, including the binding‐site residues, fall within the preferred region of the Ramachandran plot for α‐helices (Figure S8).^[29]^

**Figure 4:**
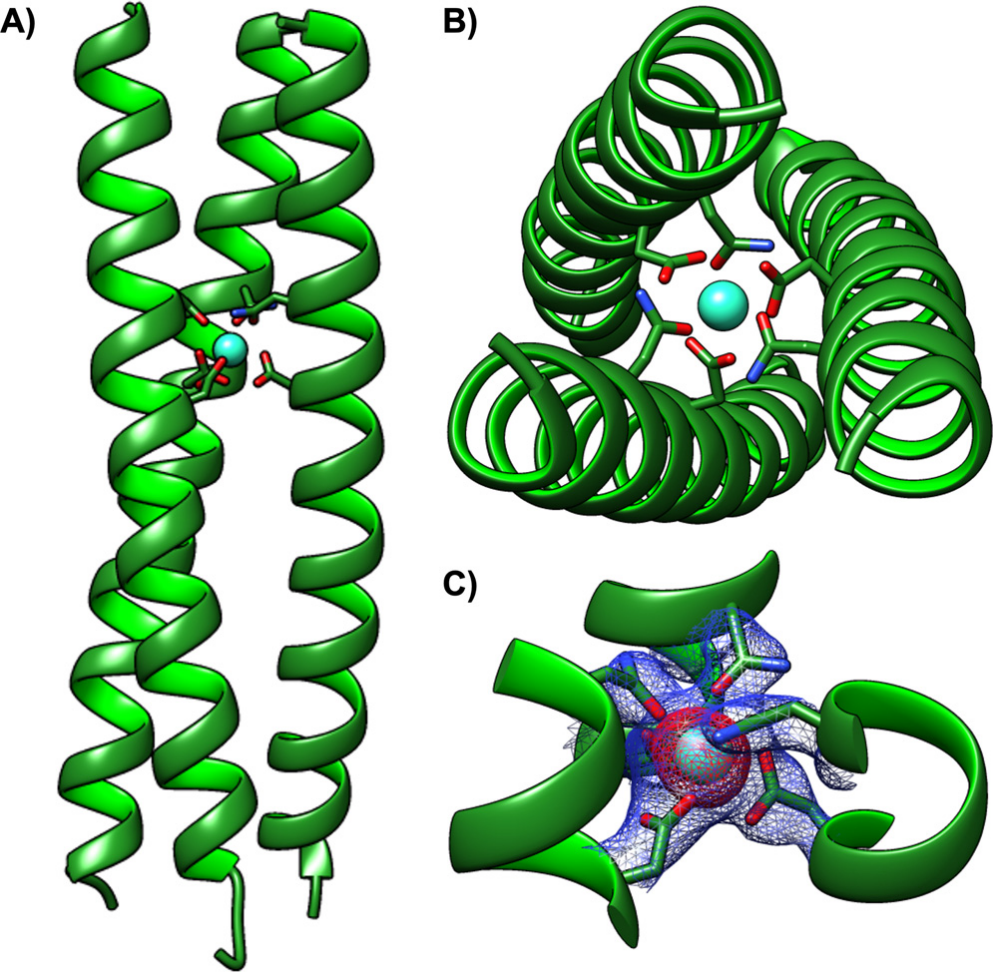
Ribbon diagrams of the Tb^3+^‐bound HC02 parallel three‐stranded coiled coil. Main‐chain atoms are shown as ribbons (N‐terminus at the top), the binding‐site Asn and Asp side chains in stick form (C green, O red, N blue) and the Tb^3+^ ion located in the binding site as a turquoise sphere. Included are A) a side‐on and B) top‐down view from the N‐terminus of the coiled coil of the full structure, and C) a close‐up of the binding site with the electron density map (blue mesh, 2.3 σ) and the Tb^3+^ anomalous scattering (red mesh, 3.5 σ) overlaid.

Consistent with the experimental evidence of an optimal radius, attempts to obtain suitable crystals with smaller and larger ions, such as Yb^3+^ and La^3+^, respectively, have so far been unsuccessful.

In view of the different experimental binding behavior of small (Yb^3+^), medium (Tb^3+^) and large (La^3+^) Ln^3+^ ions to the MB1‐2 binding site, their ability to form well folded metallocoiled coils was interrogated by circular dichroism (CD). Apo MB1‐2 is a poorly folded peptide, due to the presence of a central destabilizing (Asn‐Xxx_3_‐Asp)_3_ binding site. Ln^3+^ binding induces coiled coil formation, and does so for all three ions regardless of size. The degree of coiled coil formation, determined from the signal intensity at 222 nm, is very similar for metal titrations performed at 100 μM MB1‐2 monomer concentration (Figure S9). Despite coiled coil folding being induced largely to the same extent regardless of ionic size, the Tb^3+^ displacement experiments performed at this higher concentration of MB1‐2 retained the same size discrimination as determined at lower concentrations (see Figure S5). In contrast, notable differences are evident for the analogous CD titrations performed at the lower concentration of 10 μM MB1‐2 monomer. Binding Tb^3+^ at 10 μM MB1‐2 monomer leads to a better folded coiled coil (82±2 %), than the smaller Yb^3+^ (60±3 %) and larger La^3+^ (62±5 %), respectively (Figure S10). These observations are consistent with Tb^3+^ being the optimal size for the binding site, and therefore more effective at inducing and templating the coiled coil fold. The lack of optimal folding for the larger and smaller Ln^3+^ ions likely reflect the un‐optimised binding site for their size, leading to ineffective displacement of bound Tb^3+^. The size dependent preference in the Tb^3+^ displacement studies may reflect how well the binding sites are assembled with the metals as templates.

In conclusion, we demonstrate for the first time that designed coiled coils can discriminate between Ln^3+^ ions based on their size, and that this ability to discriminate is related to the location at which the binding‐site has been introduced. Buried sites are able to discriminate against larger ions, which are generally preferred by native lanthanide binding proteins, but also show a remarkable discrimination against smaller ions, despite the latter frequently displaying high affinities for native Ca^2+^ protein binding sites due to their high charge density.^[12]^ The (Asn‐Xxx_3_‐Asp)_3_ binding site buried in the interior of a rigid three‐stranded coiled coil, is able to overcome this, and is pre‐organized for moderately large Ln^3+^ ions (an optimal ionic radius between that of Nd^3+^ and Tb^3+^). This selectivity is structural in origin, as supported by the first crystal structure of a lanthanide binding site engineered within the hydrophobic core of a coiled coil, and with the coordination of larger and smaller Ln^3+^ ions to these sites not being suitable for optimal coiled coil folding at lower concentrations. In contrast, the flexibility associated with N‐terminal binding sites (in our design the C‐terminus site is more buried than the N‐terminus site), allow for local structural rearrangements so as to accommodate smaller, charge‐dense Ln^3+^ ions, resulting in a more promiscuous binding site. Though some degree of Ln^3+^ selectivity has previously been noted, the advantages of sites engineered into coiled coils are two‐fold: 1) the ability to discriminate or not, can be programmed into the sequence by choice of binding‐site location; and 2) the use of coiled coils of differing oligomeric states should allow for tuning of the optimal radius, and therefore generation of sites bespoke for any given Ln^3+^ ion.

This work begins to provide insight into what the design rules are for the preparation of metal binding sites capable of discriminating between very similar metal ions, which coupled with the ability to turn‐off this feature and generate almost identical promiscuous sites unable to do so, represents an exciting opportunity to be exploited in metallopeptide‐, as well as metalloprotein‐design more widely. These findings will lead to the development of functional lanthanide peptides and proteins, which harness the attractive magnetic and photophysical properties of these ions. As well as the more challenging ambition of designing a peptide or protein which features multiple orthogonal sites for distinct Ln^3+^ ions. As such we believe these findings could have implications for biotechnology and synthetic biology more widely, as well as for better understanding of native lanthanide biochemistry.

## Conflict of interest

The authors declare no conflict of interest.

## Supporting information

As a service to our authors and readers, this journal provides supporting information supplied by the authors. Such materials are peer reviewed and may be re‐organized for online delivery, but are not copy‐edited or typeset. Technical support issues arising from supporting information (other than missing files) should be addressed to the authors.

Supporting Information

Supporting Information
