# Supplementary material for: Location‐Dependent Lanthanide Selectivity Engineered into Structurally Characterized Designed Coiled Coils
Source: Angew Chem Int Ed Engl. 2021 Oct 7;60(46):24473–7. doi: 10.1002/anie.202110500 (PMC8597134; doi:10.1002/anie.202110500)
Supplement: Supplementary file 2 — Supporting Information [file ANIE-60-24473-s001.pdf]

Angewandte Chemie
Eine Zeitschrift der Gesellschaft Deutscher Chemiker

Supporting Information

Location-Dependent Lanthanide Selectivity Engineered into Structurally Characterized Designed Coiled Coils

*Louise N. Slope, Oliver J. Daubney, Hannah Campbell, Scott A. White, and Anna F. A. Peacock**

anie_202110500_sm_miscellaneous_information.pdf
anie_202110500_sm_HC02_refine_47.pdb

Supporting Information

Contents:

1. Materials and Methods
2. Figure S1 – HPLC and mass spectrum for HC02
3. Figure S2 – Representative luminescence displacement spectra for MB1-1.
4. Figure S3 – Representative luminescence displacement spectra for MB1-3.
5. Figure S4 – Luminescence displacement plots.
6. Figure S5 – Luminescence displacement plots for MB1-2 at 30 and 100 μM.
7. Figure S6 – Tb^{3+} binding CD titration into 90 μM HC02 monomer solution
8. Table S1 – Crystallographic Data Collection and Refinement Statistics
9. Table S2 – Crystallographic Tb(III)-Oxygen bond lengths
10. Figure S7 – Binding site and Tb(III) ion density maps
11. Figure S8 – Ramachandran plot
12. Figure S9 – Ln^{3+} binding CD titrations into 100 μM MB1-2 monomer solution
13. Figure S10 – Ln^{3+} binding CD titrations into 10 μM MB1-2 monomer solution
14. References

Materials and Methods, two Supplementary Tables and 10 Supplementary Figures.

1. Materials and Methods

Materials: Chemicals were used as received and purchased from; Sigma Aldrich (diethyl ether, gadolinium chloride hexahydrate, ytterbium chloride hexahydrate, erbium chloride hexahydrate, terbium chloride hexahydrate, neodymium chloride hexahydrate, lanthanum chloride heptahydrate, cerium chloride heptahydrate and 2-(N-morpholino)ethanesulfonic (MES) acid hydrate), Pepceuticals (Fmoc protected amino acids, HBTU (O-benzotriazole-N,N,N',N'-tetramethyluronium-hexafluorophosphate), synthesis grade DMF (dimethylformamide) and 20% piperidine in DMF premix), AGTC Bioproducts Ltd. (rink amide MBHA resin, NMP (N-methyl-2-pyrrolidone), DIEA (N,N-diisopropylethylamine) and DCM (dichloromethane)), Acros Organics (europium chloride hexahydrate, xylenol orange sodium salt, acetic anhydride, TIPS (triisopropylsilane) and TFA (trifluoroacetic acid)), Rathburn Chemicals Ltd. (>99.9% DMF), Fisher Scientific Ltd. (HPLC (high pressure liquid chromatography) grade water and acetonitrile, HEPES (2-[4-(2-hydroxyethyl)piperazin-1-yl]ethanesulfonic acid), urea, EDTA (ethylenediaminetetraacetic acid) and glacial acetic acid), Alfa Aesar (dysprosium chloride hexahydrate), VWR International (lutetium chloride hexahydrate) and Strem Chemicals UK Ltd. (praseodymium chloride heptahydrate and samarium chloride hexahydrate).

Peptide Synthesis, Characterization and Purification: The peptides used in this work (see Table 1) were synthesized, purified and characterized as reported previously.[1]

UV-visible spectroscopy: UV-visible spectra were recorded on a Shimadzu 1800 UV Spectrophotometer, recorded in single beam mode with a medium scan speed, slit width of 1.0 nm, data interval of 1.0 nm and scan range 420-260 nm (Trp), using a 1 cm pathlength, 700 μL quartz cuvette.

Solution Preparation: The concentration of freshly prepared peptide stock solutions in MilliQ water, were deduced from the absorption of the Trp residue at 280 nm ($\varepsilon_{280} = 5690\ M^{-1}\ cm^{-1}$) in 7

M aqueous urea, performed in triplicate for accuracy and left for 10 mins prior to quantitative UV determination. The concentrations of freshly prepared stock solutions of $LnCl_3$ (~1 mM) in MilliQ water, were determined in triplicate using a xylenol orange indicator and EDTA titration with Ln^{3+} standard solutions, following a procedure previously reported by Fedeli and co-workers.[2]

Luminescence: Luminescence data for Tb^{3+} emission signals were acquired on an Edinburgh Instruments Fluorescence FL920 system with a 450 W Xenon arc lamp and a Hamamatsu R928 photomultiplier tube. The emission monochromator was fitted with two interchangeable gratings blazed at 500 nm and 1200 nm and the data were collected using F900 spectrometer analysis software. A 455 nm-long pass filter was used, the solutions were excited at 280 nm and the emission scanned in the range 455-700 nm using an excitation slit width of 5 nm and an emission slit width of 5 nm with a 0.5 sec dwell time, following a previously reported procedure.**Error! Bookmark not defined.**

Displacement Studies: Experiments were performed in a 1 cm pathlength quartz cuvette. Emission spectra of 30 μM peptide monomer solution in 10 mM HEPES buffer pH 7.0, were recorded following 15 minutes, 24 hours and 72 hours equilibration. The emission profiles of the five peptides were recorded in three sets: 1) the absence of Tb^{3+}; 2) in the presence of one equivalence (per trimer) of Tb^{3+} only; and 3) in the presence of one equivalence of Tb^{3+} and an additional equivalence of a second competing Ln^{3+} ion, where Ln^{3+} can be Lu^{3+}, Yb^{3+}, Er^{3+}, Dy^{3+}, Gd^{3+}, Eu^{3+}, Sm^{3+}, Nd^{3+}, Pr^{3+}, Ce^{3+} or La^{3+}. The Tb^{3+} signal intensity (integrated from 530-560 nm) was measured, with loss of the Tb^{3+} signal associated with Tb^{3+} displacement by the second Ln^{3+}. These experiments were performed in triplicate, an average taken and the standard deviation reported. The same experiments were also carried out in 30 mM MES buffer pH 5.5, and for 100 μM MB1-2 monomer solutions in 10 mM HEPES buffer pH 7.0, following 72 hours equilibration.

Circular Dichroism: CD spectra were recorded on a Jasco J-715 Spectropolarimeter in a 1 cm pathlength quartz cuvette for 10 μM MB1-2 monomer solutions and in a 1 mm pathlength quartz cuvette for 100 μM MB1-2 or 90 μM HC02 monomer solutions. The optical chamber was purged with nitrogen and kept under a nitrogen atmosphere throughout the duration of the experiments. 1 mM stock solutions of $LnCl_3$ (where Ln^{3+} is either Tb^{3+}, La^{3+} or Yb^{3+}) were titrated into 10 μM peptide monomer in 5 mM HEPES buffer pH 7.0 (10 mM HEPES buffer pH 7.0 for 100 μM MB1-2 or 90 μM HC02 monomer solutions), as previously reported.[3] All solutions were left to equilibrate for 10 minutes before recording spectra, and the observed ellipticity converted into molar ellipticity, with the helical content calculated as the percentage folded, based on the theoretical maximum ellipticity as reported by Scholtz et al.[4]

Crystallography: Crystals of HC02-Tb(III) were grown using the sitting-drop vapor-diffusion technique at 18 °C with a drop consisting of 3 μL peptide stock solution (16 mg mL^{-1} HC02, 6 mM $TbCl_3$ and 5 mM $Zn(OAc)_2$) mixed with 3 μL precipitant solution (0.21 M NH_4Cl, 25% PEG3350). Rectangular plate-like crystals belonging to the space group *H3* grew within a few weeks to a maximum dimension of approximately 0.6 × 0.2 mm. Crystals were transferred to a stabilization solution based on the mother liquor and were then cryo-protected by increasing the concentration of ethylene glycol (up to 25%) before flash-cooling and storage in a liquid nitrogen cooled dewar.

The HC02-Tb^{3+} data set was collected at Diamond Light Source utilizing beam i04-1. The data-set was indexed and integrated with iMosflm,[5] reduced with POINTLESS,[6] scaled using AIMLESS and structure amplitudes were calculated using CTRUNCATE.[7,8] The Tb^{3+} ions provided heavy metal anomalous signals, from which their positions were located and phases determined using Phaser within the Phenix pipeline.[9,10] This resulted in a clearly interpretable electron density map with a particularly clear lanthanide binding site. The difference in

anomalous scattering between the two different metal ions present, Tb^{3+} and Zn^{2+}, allowed for their assignment.

Coot was used for model adjustment and manual refinement while phenix.refine with ML targets was used for intervals of automated refinement.[11,12] The resulting structure was refined to 2.10 Å resolution. Data-collection, refinement and validation statistics are presented in Table S1. The atomic coordinates and structure factors for HC02-Tb^{3+} have been deposited in the Protein Data Bank with the ID code 7P3H. Figures were generated in UCSF Chimera.[13]

2. Figure S1:

A)

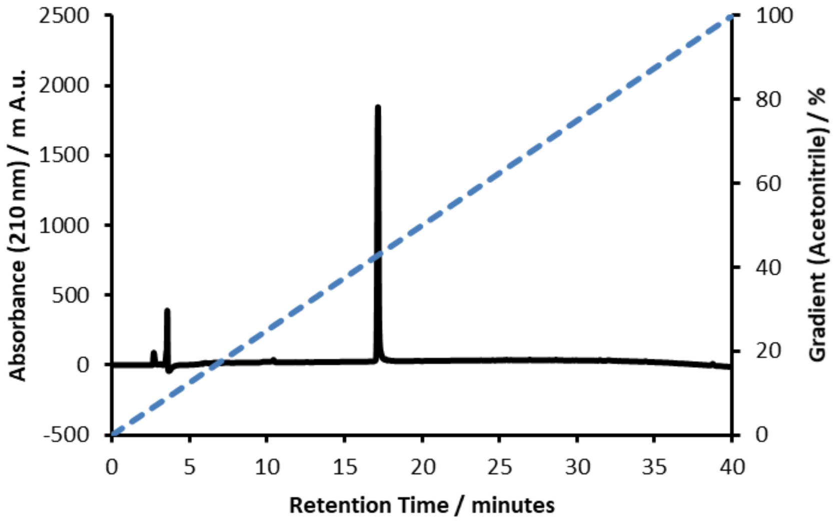

B)

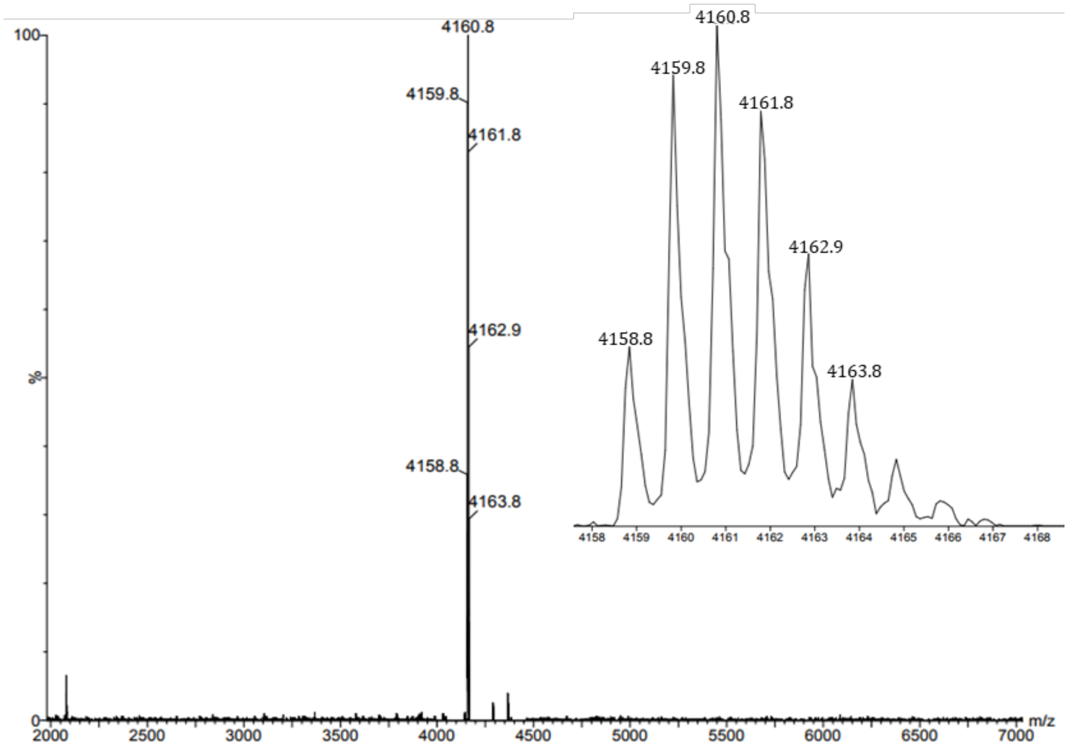

Figure S1. A) C18-analytical HPLC trace of purified HC02 using a linear gradient from 0-100% acetonitrile in water with 0.05% TFA (blue dashed line) over 40 minutes. B) MALDI mass spectrum of purified HC02 with inset showing the isotope distribution of the +1 charge peak.

3. Figure S2:

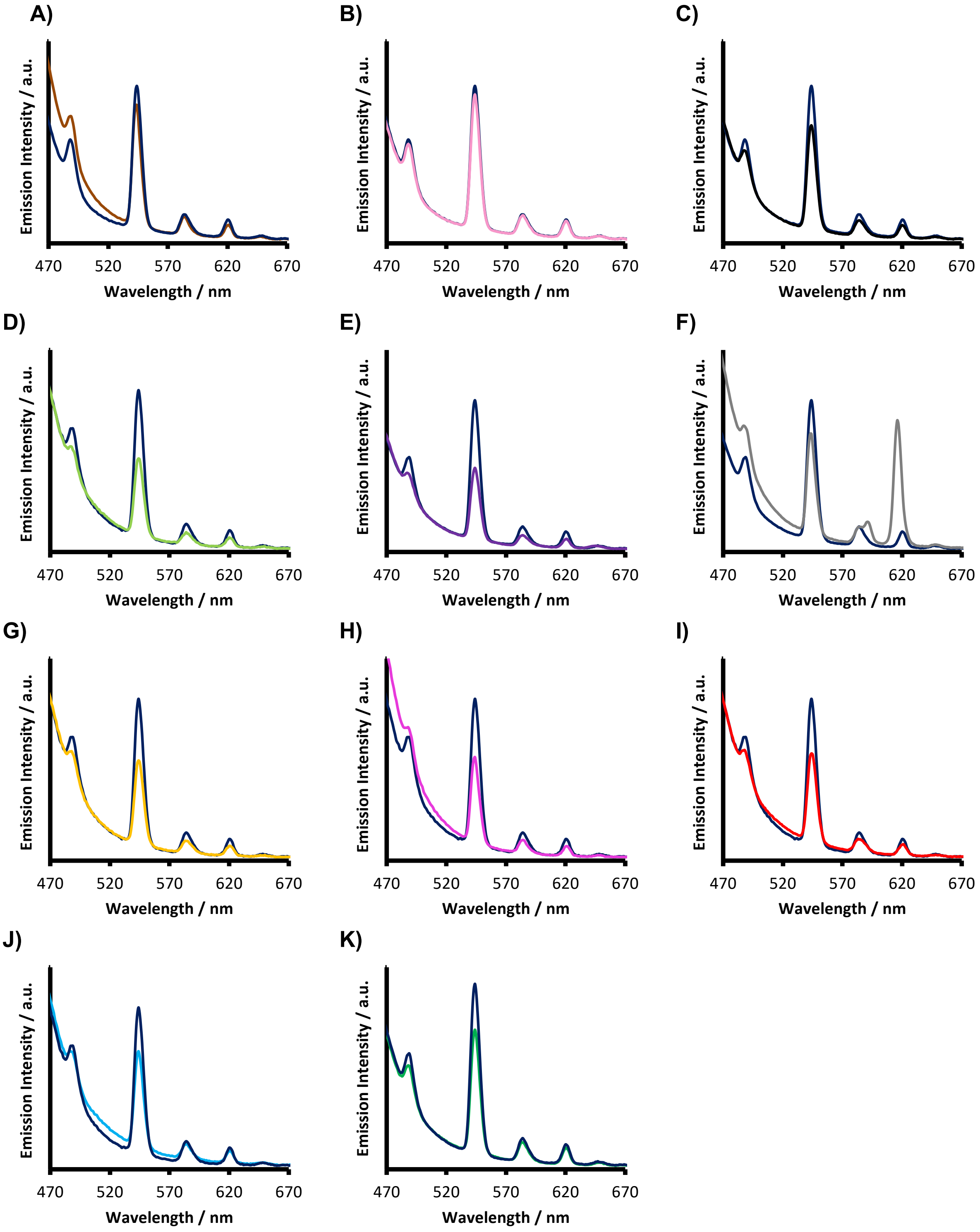

Figure S2. Representative Tb^{3+} (10 μM) emission spectra recorded in the presence of MB1-1 (30 μM) in 10 mM HEPES buffer pH 7.0 after 72 hours equilibration (λ_{ex} = 280 nm), in the absence (dark blue line) and presence of 1 equivalent of a second competing Ln^{3+} ion, where Ln^{3+} is either

A) La^{3+} (brown), B) Ce^{3+} (pink), C) Pr^{3+} (black), D) Nd^{3+} (light green), E) Sm^{3+} (dark purple), F) Eu^{3+} (grey), G) Gd^{3+} (orange), H) Dy^{3+} (light purple), I) Er^{3+} (red), J) Yb^{3+} (light blue) or K) Lu^{3+} (dark green).

4. Figure S3

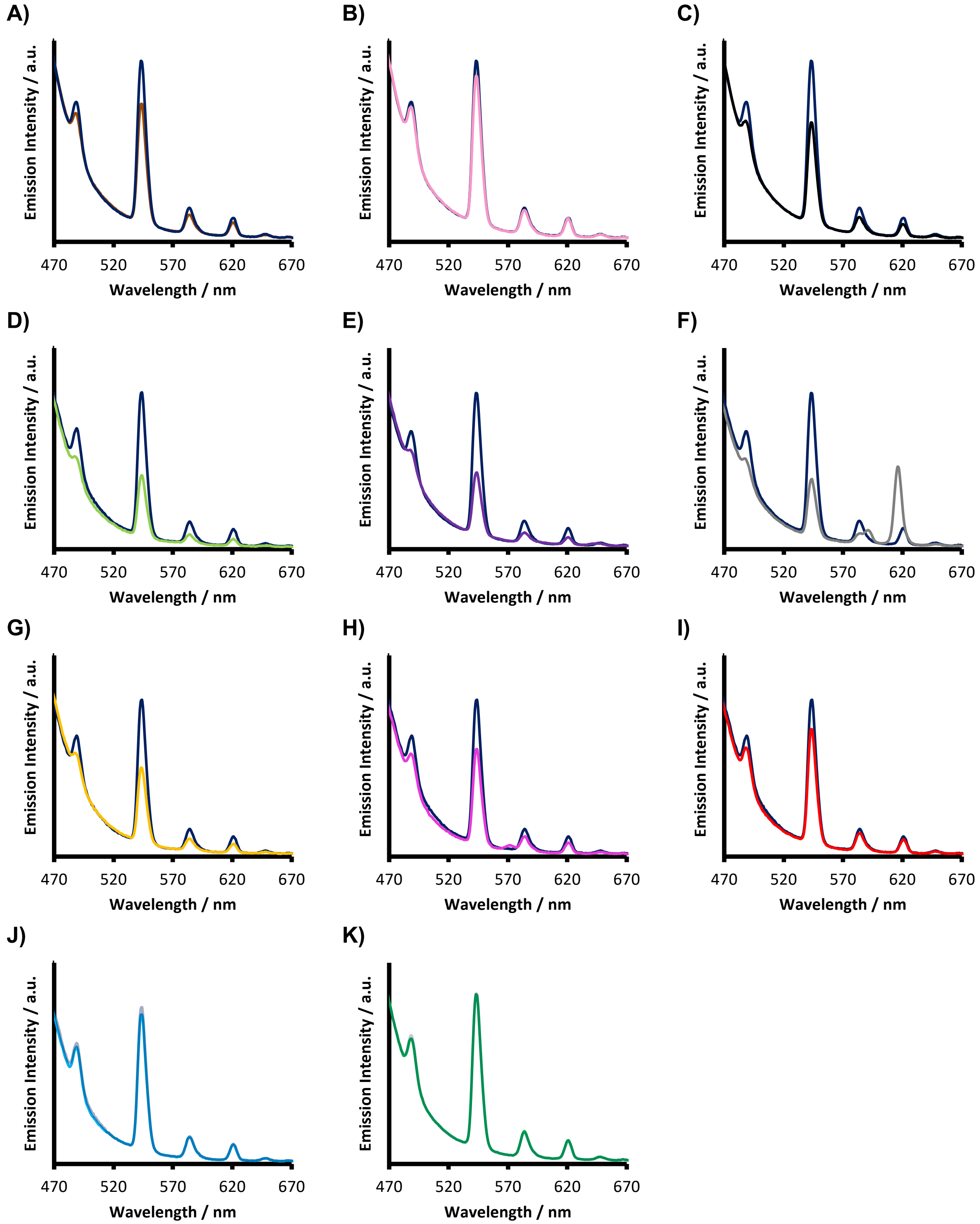

Figure S3. Representative Tb^{3+} (10 μM) emission spectra recorded in the presence of MB1-3 (30 μM) in 10 mM HEPES buffer pH 7.0 after 72 hours equilibration (λ_{ex} = 280 nm), in the absence

(dark blue line) and presence of 1 equivalent of a second competing Ln^{3+} ion, where Ln^{3+} is either A) La^{3+} (brown), B) Ce^{3+} (pink), C) Pr^{3+} (black), D) Nd^{3+} (light green), E) Sm^{3+} (dark purple), F) Eu^{3+} (grey), G) Gd^{3+} (orange), H) Dy^{3+} (light purple), I) Er^{3+} (red), J) Yb^{3+} (light blue) or K) Lu^{3+} (dark green).

5. Figure S4.

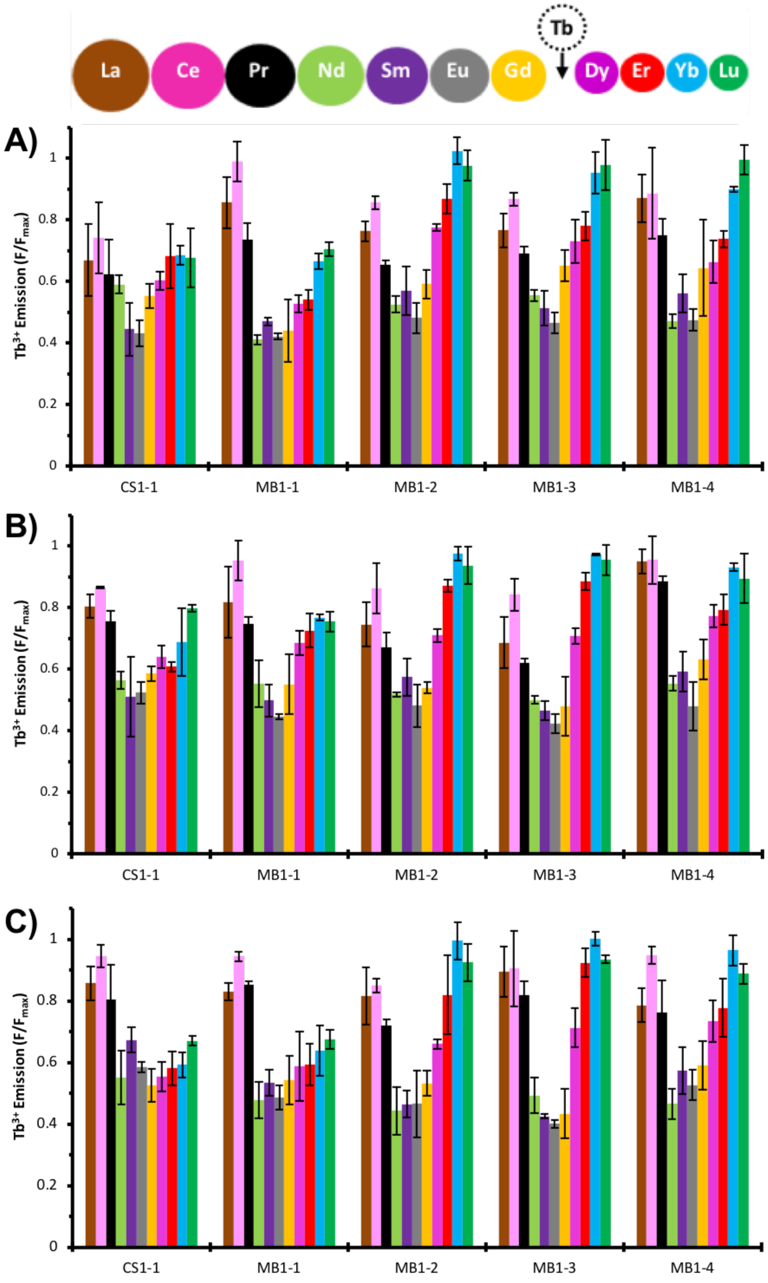

Figure S4. Luminescence Tb^{3+} displacement plots for 10 μM Tb^{3+} and 30 μM peptide monomer in the presence of 10 μM competing Ln^{3+} ion, in 10 mM HEPES buffer pH 7.0 following A) 15 minutes and B) 24 hours equilibration, and C) in 30 mM MES buffer pH 5.5 following 72 hours equilibration. Data are based on the integration of the 545 nm Tb^{3+} emission peak, for experiments performed in triplicate, and bars represent the standard deviation. Spheres are shown as an indication of the change in lanthanide size across the series.

6. Figure S5:

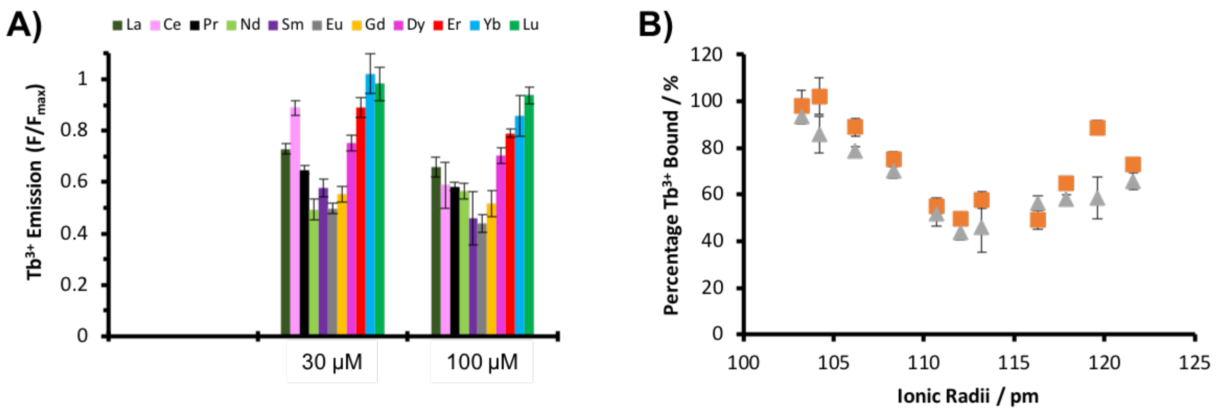

Figure S5. Luminescence Tb^{3+} displacement plots for 30 µM and 100 µM MB1-2 monomer in the presence of 1/3 equivalence Tb^{3+} and competing Ln^{3+} ion, in 10 mM HEPES buffer pH 7.0 following 72 hours equilibration. Data are based on the integration of the 545 nm Tb^{3+} emission peak, for experiments performed in triplicate, and bars represent the standard deviation. Displacement profiles are shown as A) bar charts, or B) as a function of ionic radius (30 µM MB1-2 orange squares; 100 µM MB1-2 grey triangles).

7. Figure S6:

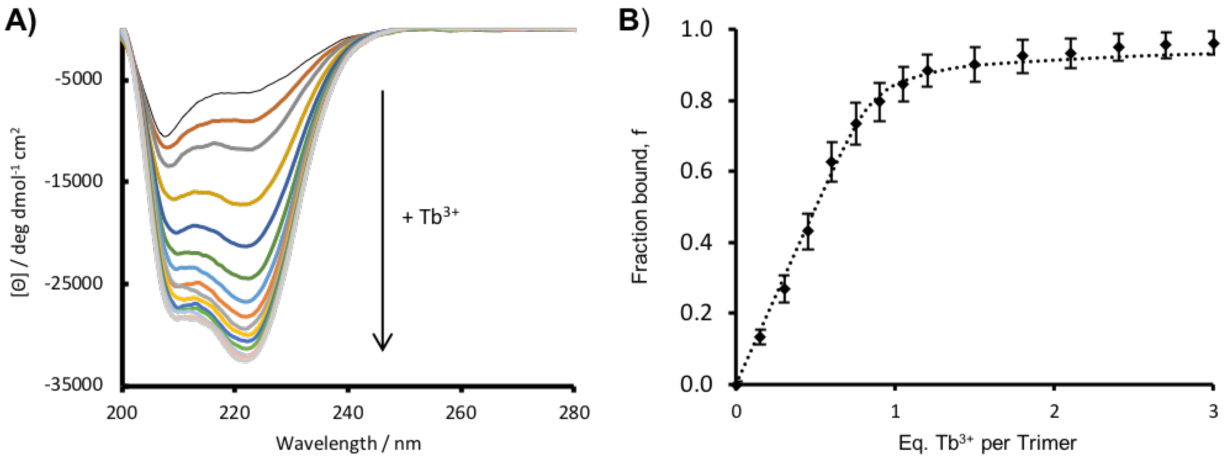

Figure S6. A) $TbCl_3$ titration into 90 μM HC02 monomer in 10 mM HEPES buffer pH 7.0, monitored by CD. B) Plot of fraction of peptide bound (based on molar ellipticity at 222 nm) as a function of Tb^{3+} equivalents per trimer. Data for experiments performed in triplicate, and bars represent the standard deviation. The dotted Line represents the best fit to a monomer-to-trimer model, as previously reported, yielding a log K_a of 5.13 ± 0.11.**Error! Bookmark not defined.**

8. Table S1. Data Collection and Refinement Statistics:

	HC02-Tb(III)
**Crystal Properties**	
Space group	R3:H
Unit cell parameters (Å)	
a = *b*	103.0
c	46.9
**Data Collection**	
Wavelength (Å)	0.9282
No. of observations	49925 (5130)
No. of unique reflections	10820 (1075)
Completeness (%)	99.9 (99.7)
Multiplicity	4.6 (4.8)
Mean I/σ(I)	13.2 (2.76)
R_{merge}(%)	0.076 (0.450)
$CC_{1/2}$	0.99 (0.93)
Resolution range (Å)	41.5 - 2.10 (2.175 - 2.10)
Wilson *B* (Å^2)	54.0
**Refinement**	
No. of non-H atoms	962
No. of waters	68
R_{work}/R_{free}†	0.210/0.234
CC_{work}/CC_{free}	0.952/0.939
R.m.s.d. bonds (Å) / angles (°)	0.009/0.90
Ramachandran‡	99/1/0/0
Average *B* (Å^2)	86.9
PDB code	7P3H

† R_{free} was calculated for a random set of 5% of reflections that were not used in the refinement.
‡ Number of non-glycine, nonproline amino acids in the core/allowed/generously allowed/disallowed regions of the Ramachandran plot.
Values in parentheses apply to data in the highest resolution shell.

9. Table S2. Terbium-Oxygen distances in HC02-Tb(III) crystal structure:

**Residue / Atom**[a]	**Chain ID**	**Terbium-Oxygen Distance / Å**
Asp(16) / OD1	A	2.4
OD2		2.4
Asp(16) / OD1	B	2.4
OD2		2.3
Asp(16) / OD1	C	2.6
OD2		2.5
Asn(12)	A	2.3
Asn(12)	B	2.3
Asn(12)	C	2.4

[a] Numbers in parenthesis correspond to the sequence position of the residue.

10. Figure S7:

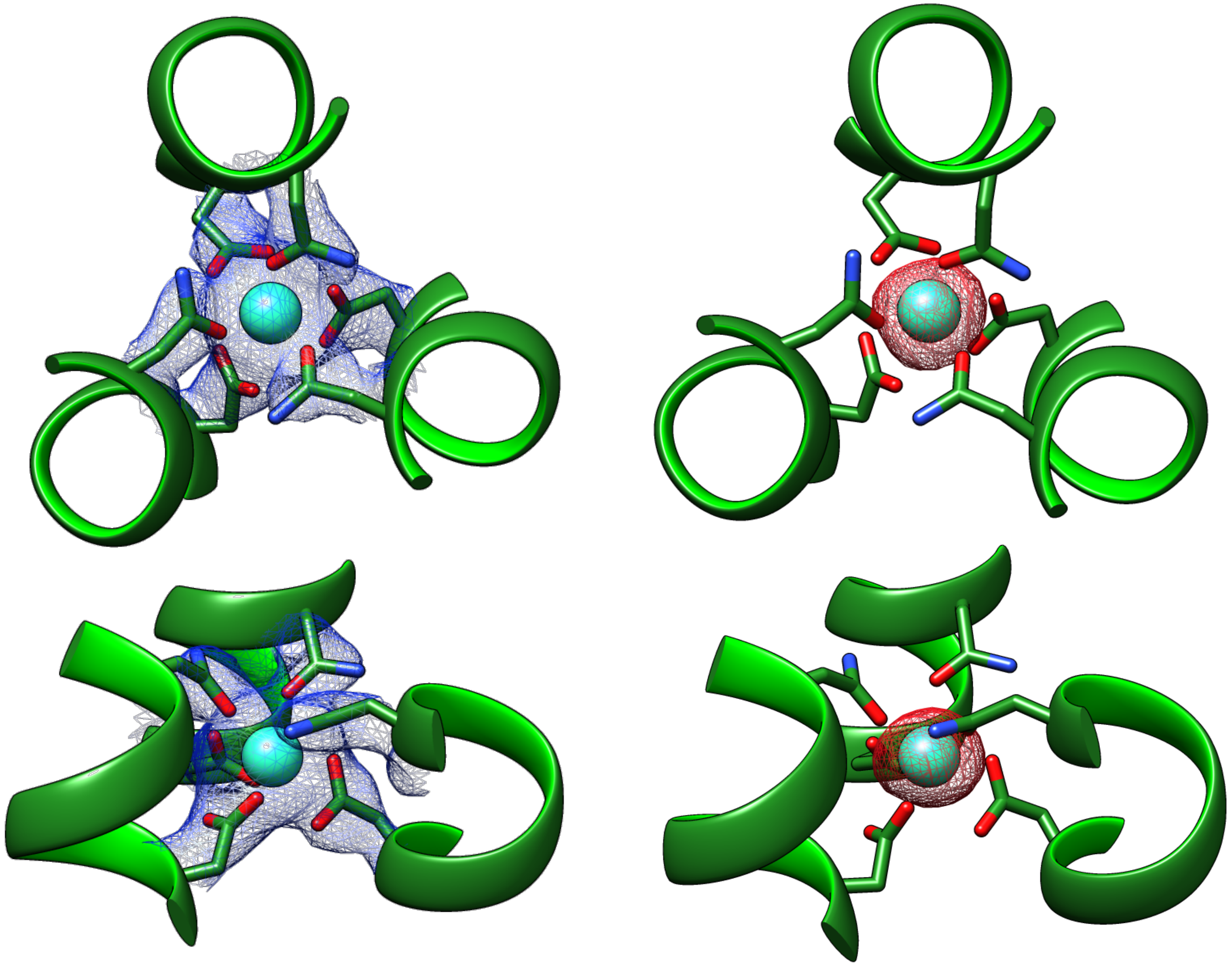

Figure S7. A) Density map computed from the observed HC02 data-set (blue mesh, 2.3 σ), and the Tb(III) anomalous scattering (red mesh, 3.5 σ) overlaid with the crystallographic structure of the binding site. The placement and intensity of the density clearly indicates the presence of six ligands around a single terbium atom at the centre of the designed binding site.

11. Figure S8:

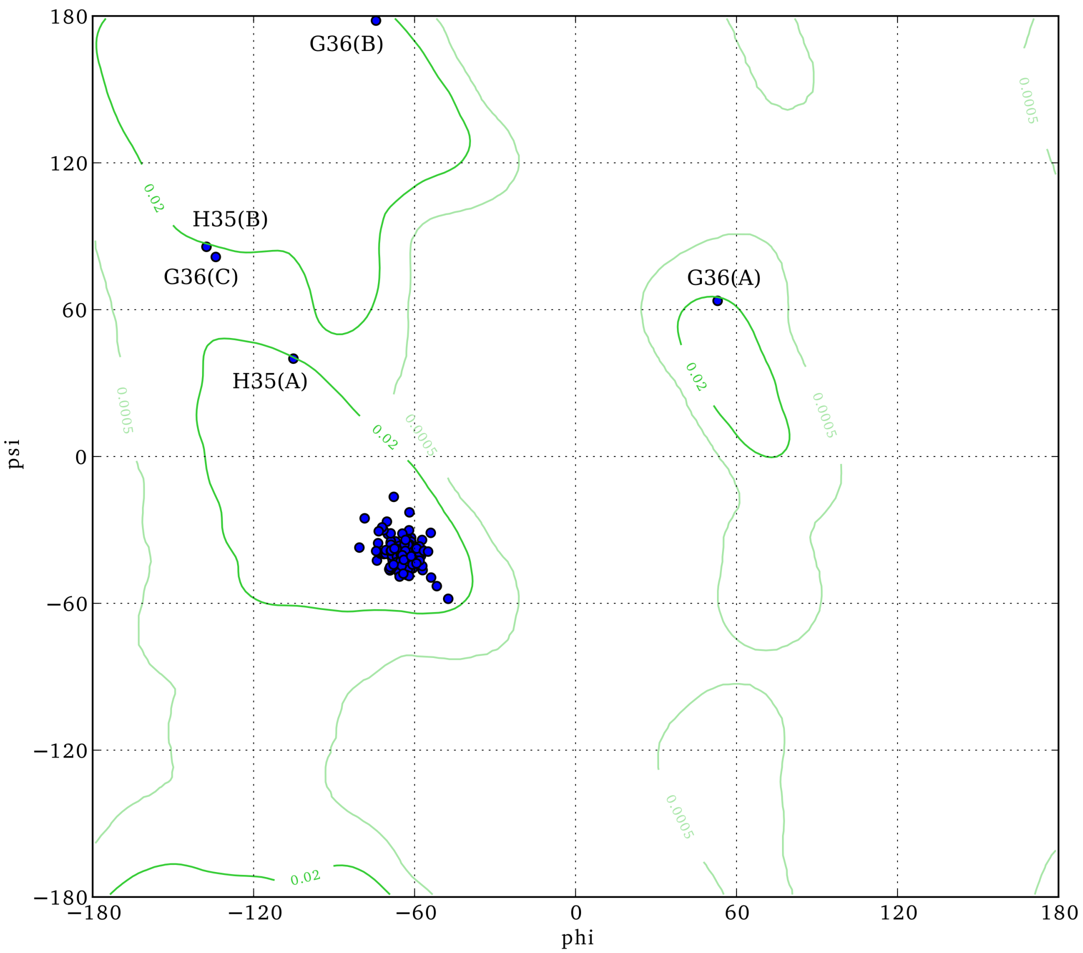

Figure S8. Ramachandran plot for HC02-Tb(III) prepared using Chimera, illustrating that residues 3-33 in the peptide are well-folded α-helices. Contour lines demarcate the zones with less than 2% and 0.05% probability when compared to a reference set of high-resolution protein structures.[S14] The terminal non-Gly residues have some deviation from the α-helical structure (allowed outliers) due to coordination with external metal cations.

12. Figure S9:

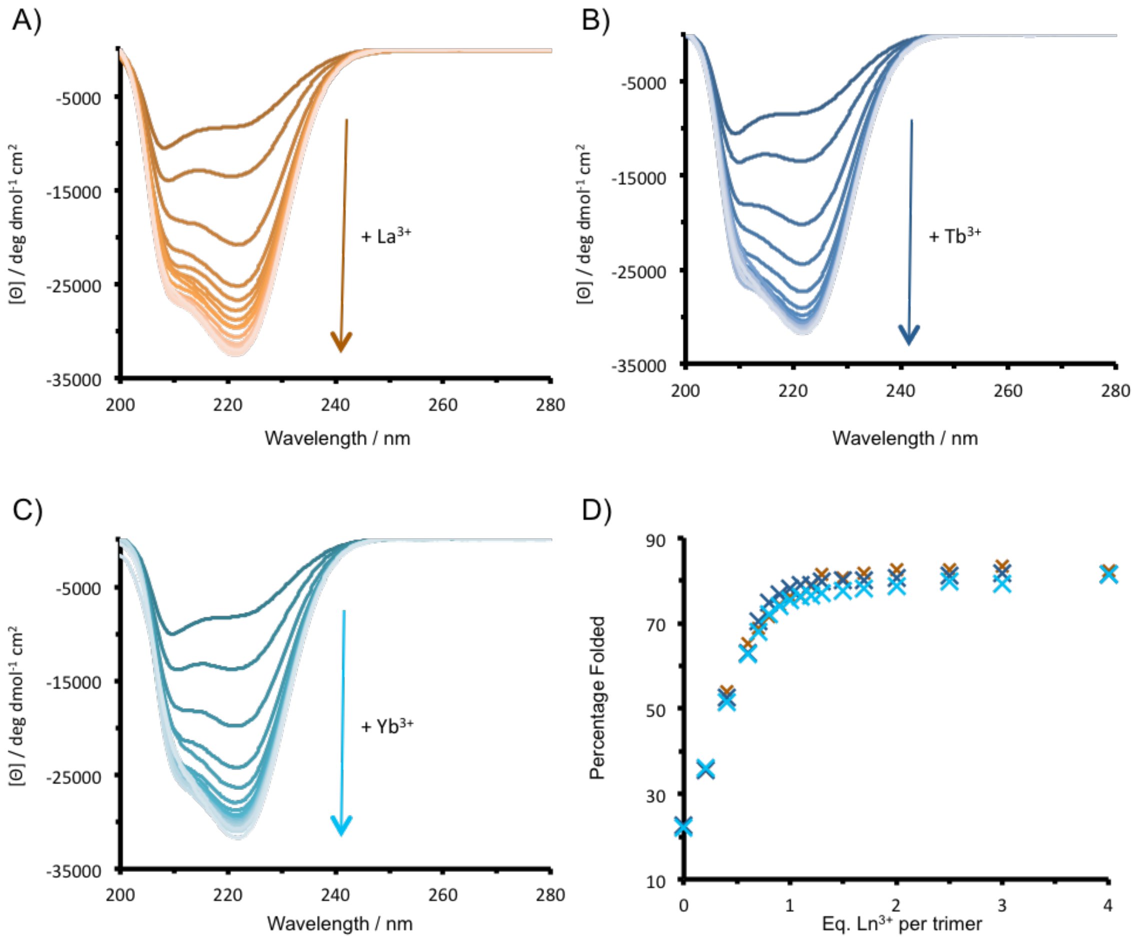

Figure S9. Metal binding titrations monitored by CD on addition of A) La^{3+} (brown), B) Tb^{3+} (dark blue) and C) Yb^{3+} (light blue), to a 100 µM MB1-2 monomer solution in 10 mM HEPES buffer pH 7.0. D) Plot of percentage folded (based on molar ellipticity at 222 nm) as a function of Ln^{3+} equivalents per trimer. The degree of coiled coil formation is similar for metal titrations performed at 100 µM MB1-2 monomer concentration.

13. Figure S10:

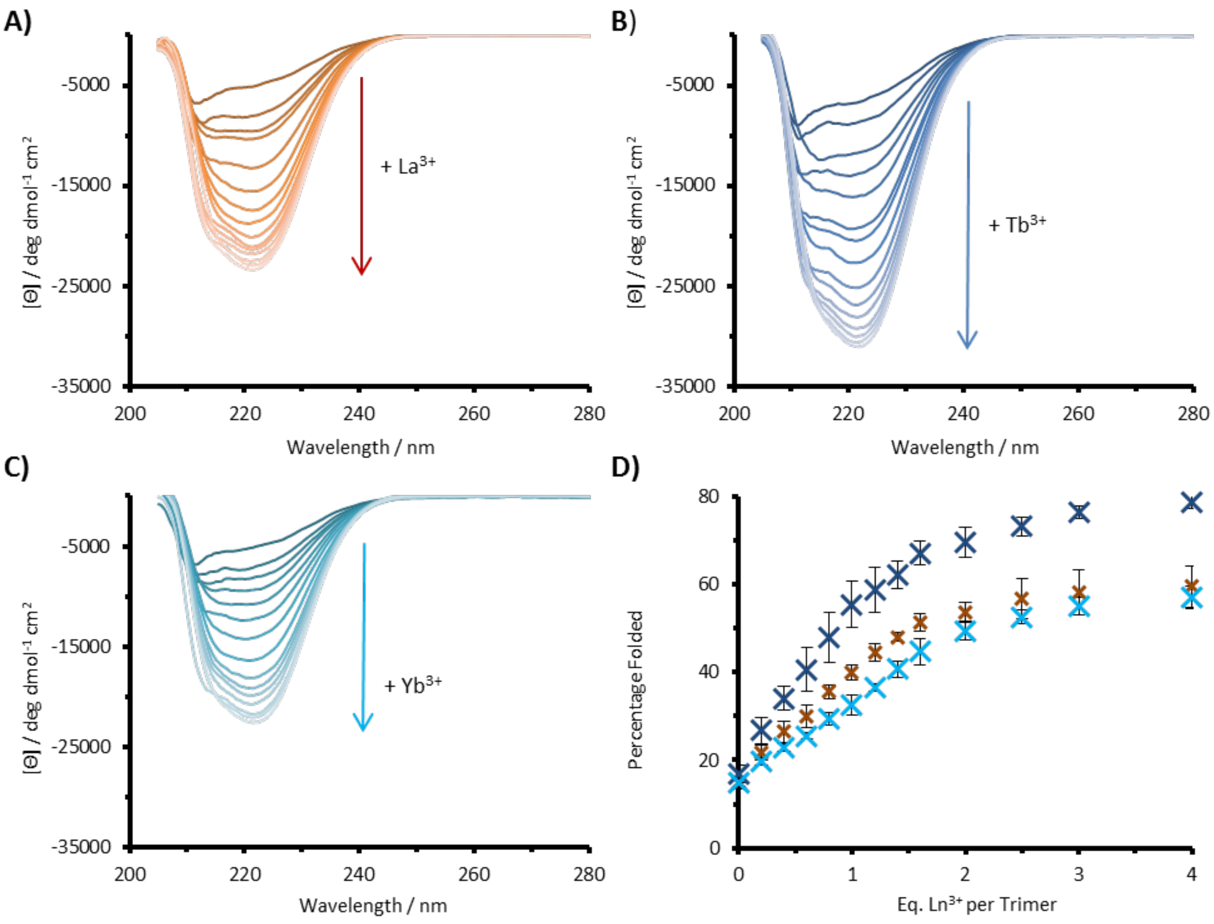

Figure S10. Representative metal binding titrations monitored by CD on addition of A) La^{3+} (brown), B) Tb^{3+} (dark blue) and C) Yb^{3+} (light blue), to a 10 µM MB1-2 monomer solution in 5 mM HEPES buffer pH 7.0. D) Plot of percentage folded (based on molar ellipticity at 222 nm) as a function of Ln^{3+} equivalents per trimer. Data for experiments performed in triplicate, and bars represent the standard deviation. Though Ln^{3+} binding induces coiled coil formation, for all three ions regardless of size, it does so to a differing degree. Binding Tb^{3+} leads to a better folded coiled coil (82±2%), than the smaller Yb^{3+} (60±3%) and larger La^{3+} (62±5%), respectively. These observations are consistent with Tb^{3+} being the optimal size for the binding site, and therefore more effective at inducing and templating the coiled coil fold.

14. Supporting Information References

[S1] M. R. Berwick, L. N. Slope, C. F. Smith, S. M. King, S. L. Newton, R. B. Gillis, G. G. Adams, A. J. Rowe, S. E. Harding, M. M. Britton, A. F. A. Peacock, *Chem. Sci.,* **2016**, *7*, 2207-2216.

[S2] A. Barge, G. Cravotto, E. Gianolio, F. Fedeli, *Contrast Media Mol. Imaging,* **2006**, *1*, 184-188.

[S3] M. R. Berwick, D. J. Lewis, A. W. Jones, R. A. Parslow, T. R. Dafforn, H. J. Cooper, J. Wilkie, Z. Pikramenou, M. M. Britton, A. F. A. Peacock, *J. Am. Chem. Soc.* **2014**, *136*, 1166-1169.

[S4] J. K. Myers, C. N. Pace, J. M. Scholtz, *Proc. Natl. Acad. Sci. U.S.A.* **1997**, *94*, 2833-2837.

[S5] a) T. G. G. Battye, L. Kontogiannis, O. Johnson, H. R. Powell, A. G. W. Leslie, *Acta Crystallogr.*, *Sect. D: Biol. Crystallogr.* **2011**, *67*, 271-281; b) M. D. Winn, C. C. Ballard, K. D. Cowtan, E. J. Dodson, P. Emsley, P. R. Evans, R. M. Keegan, E. B. Krissinel, A. G. W. Leslie, A. McCoy, S. J. McNicholas, G. N. Murshudov, N. S. Pannu, E. A. Potterton, H. R. Powell, R. J. Read, A. Vagin, K. S. Wilson, *Acta Crystallogr.*, *Sect. D: Biol. Crystallogr.* **2011**, *67*, 235-242.

[S6] a) P. R. Evans, *Acta Crystallogr.*, *Sect. D: Biol. Crystallogr.* **2006**, *62*, 72-82; b) P. R. Evans, *Acta Crystallogr.*, *Sect. D: Biol. Crystallogr.*, **2011**, *67*, 282-292.

[S7] P. R. Evans, G. N. Murshudov, *Acta Crystallogr.*, *Sect. D: Biol. Crystallogr.* **2013**, *69*, 1204-1214.

[S8] a) S. French, K. Wilson, *Acta Crystallogr.*, *Sect A: Found. Crystallogr.*, **1978**, *34*, 517-525; b) J. E. Padilla, T. O. Yeates, *Acta Crystallogr.*, *Sect. D: Biol. Crystallogr.* **2003**, *59*, 1124-1130; c) A. N. Popov, G. P. Bourenkov, *Acta Crystallogr.*, *Sect. D: Biol. Crystallogr.* **2003**, *59*, 1145-1153.

[S9] A. J. McCoy, R. W. Grosse-Kunstleve, P. D. Adams, M. D. Winn, L. C. Storoni, R. J. Read, *J. Appl. Crystallogr.* **2007**, *40*, 658-674.

[S10] P. D. Adams, R. W. Grosse-Kunstleve, L. –W. Hung, T. R. Ioerger, A. J. McCoy, N. W. Moriarty, R. J. Read, J. C. Sacchettini, N. K. Sauter, T. C. Terwilliger, *Acta Crystallogr.*, *Sect. D: Biol. Crystallogr.* **2002**, *58*, 1948-1954.

[S11] P. Emsley, K. Cowtan, *Acta Crystallogr.*, *Sect. D: Biol. Crystallogr.* **2004**, *60*, 2126-2132.

[S12] P. V. Afonine, R. W. Grosse-Kunstleve, P. D. Adams, *CCP4 Newsl. Protein Crystallogr.* **2005**, *42*, 8.

[S13] E. F. Pettersen, T. D. Goddard, C. C. Huang, G. S. Couch, D. M. Greenblatt, E. C. Meng, T. E. Ferrin, *J. Comput. Chem.* **2004**, *25*, 1605-1612.

[S14] S. C. Lovell, I. W. Davis, W. B. 3rd Arendall, P. I. de Bakker, J. M. Word, M. G. Prisant, J. S. Richardson, D. C. Richardson, *Proteins,* **2003**, *50*, 437-450.
